# The Immunotherapeutic Effect of SIRP*α*-Silenced DCs against Cervical Cancer

**DOI:** 10.1155/2020/1705187

**Published:** 2020-01-07

**Authors:** Xiaojie Li, Wenying Zhou, Yanlan Liang, Changzhi Xu, Zhizhi Xie, Jiayin Liang, Bo Hu

**Affiliations:** Department of Laboratory Medicine, The Third Affiliated Hospital, Sun Yat-sen University, Guangzhou 510630, China

## Abstract

Signal regulatory protein *α* (SIRP*α*), a transmembrane protein that is predominantly expressed in dendritic cells (DCs) or macrophages, interacts with CD47 that is overexpressed in almost all types of tumor cells. The interaction between SIRP*α* and CD47 leads to a negative signal that prevents the phenotypic and functional maturation of DC and inhibits phagocytosis. The SIRP*α* knockdown in DCs that were pulsed with a modified HPV16E7 (HPV16mE7) protein with enhanced antigenicity and reduced transformation activity results in increased cytokine (TNF-*α*/IL-12/IL-6) secretion, IFN-*γ* secretion by T lymphocytes, and *in vitro*/*in vivo* tumoricidal activity against cervical cancer cells. Taken together, these results suggest that SIRP*α*-silenced DC vaccination presented potential therapeutic implications against cervical cancer.

## 1. Introduction

SIRP*α*, an inhibitory transmembrane receptor expressed especially abundant in macrophages and DCs, when engaged by its ligand CD47 which is a widely distributed membrane protein, transmits a “do not eat me signal” to the macrophages and delivers a negative signal to DCs, inhibiting phagocytosis of CD47-expressing cells and preventing the phenotypic and functional maturation of DCs [1–5]. The extracellular region of SIRP*α* comprises 3 Ig-like domains, and the cytoplasmic region contains immunoreceptor tyrosine-based inhibition motifs that bind and activate the protein tyrosine phosphatases SHP-1/SHP-2, thus inhibiting signaling through various receptor tyrosine kinases and cytokine receptors [6–9].

SIRP*α* interacts with its ligand, CD47, which is also a member of the Ig superfamily, with such interaction triggering the tyrosine phosphorylation of SIRP*α*. The interaction between SIRP*α* and CD47 on DCs counteracts the phenotypic and functional maturation of DC by inhibiting cytokine production such as IL-12/TNF-*α*/IFN-*γ* [2, 10–12]. Among these cytokines, IL-12 plays a key role in inducing IFN-*γ* production through enhancing NK cell cytotoxicity and promoting cytotoxic T cell development [13]. Attributed to their capacity to produce IL-12, DCs are critically positioned to initiate the Th1 immune response [14]. The interaction between SIRP*α* on DCs with CD47 on T cells is important for regulating the priming of naive T cells, which then differentiate into T helper cells, or induction of antigen-specific cytotoxic T cell responses by DCs [15]. Current focus on immunotherapy had been targeted toward inhibiting CD47-SIRP*α* interaction via anti-CD47 or anti-SIRP*α* antibodies [16].

As the professional antigen-presenting cells (APC), DCs are key immunity regulators to induce T cell activation as well as tolerance. The terminally differentiated mature DC can proficiently induce the development of T effectors, while immature and semimature DCs induce and maintain immune tolerance [17]. As a key negative regulator of immunity, the transcription factor Signal Transducer and Activators of Transcription-3 (STAT3) signaling is linked to DC immature phenotype, production of IL-10, and tolerance induction. The engagement of CD47 with SIRP*α* activates STAT3 signal pathway thus inhibiting DC maturation [18, 19]. CD47 expression is confirmed on nearly all cancer cells from every primary and xenograft patient tumor sample. CD47 is also overexpressed on tumor samples [20]. Targeting the CD47-SIRP*α* signaling axis is the promising strategy for cancer treatment [21, 22]. Here, in this report, SIRP*α* in mouse bone marrow- (BM-) derived DCs was knocked down by shRNA which was carried by a recombinant adenovirus. And then, the generated DCs were pulsed with a modified HPV16E7 (HPV16mE7) protein with enhanced antigenicity and reduced transformation activity [23]. The phenotypic and functional maturation as well as the immunotherapeutic effect of mE7-pulsed DCs with silenced SIRP*α* on the allogeneic cervical tumor mouse models was investigated.

## 2. Materials and Methods

### 2.1. Cells and Reagents

TC-1 and HEK293 cell lines were purchased from American Type Culture Collection (ATCC). Cells were maintained in the DMEM or RPMI-1640 culture media (Gibco, Life Technologies, USA) supplemented with 10% (*v*/*v*) fetal bovine serum (FBS) (HyClone Laboratories, US). rhGM-CSF and rmIL-4 were purchased from PeproTech Inc. LPS was a product of Sigma. pShuttle-2 and pAdeno-X vectors were acquired from BD Clontech. Adeno-X Rapid Titer kits were products of BD Bioscience. Rabbit anti-mouse CD80-PE monoclonal antibody, Rabbit anti-mouse CD40-FITC monoclonal antibody, Rabbit anti-mouse CD83-PE monoclonal antibody, Rabbit anti-mouse CD86-PE monoclonal antibody, and Rabbit anti-mouse CD1a-FITC monoclonal antibody were products from Santa Cruz Co., Ltd. Female C57BL/6 mice (16 to 22 g, 6 to 8 weeks of age) raised under SPF circumstance were purchased from the Guangzhou Traditional Chinese Medicine University. All animal studies were conducted in accordance with the *Guide for the Care and Use of Laboratory Animals*.

### 2.2. Recombinant Adenovirus Vector Construction

The interference fragment of (F) 5′-GATCCCTACCTGAGTTCAGTGAAGGTGACTCAGCCTGAAGGAACTCAGGTAGTTTTTTG-3′ and (R) 5′-AATTCAAAAAACTACCTGAGTTCCTTCAGGCTGAGTCACCTTCACTGAACTCAGGTAGG-3′ and the control interference fragment of (F) 5′-GATCCTCACAACCTCCTAGAAAGAGTAGATTGTACTACACAAAAGTACTATGTTTTTTG-3′ and (R) 5′-AATTCAAAAAACATAGTACTTTTGTGTAGTACAATCTACTCTTTCTAGGAGGTTGTGAG were cloned into pShuttle-2 and then inserted into pAdeno-X vector [23–26]. The recombinant pAdeno-X vectors were verified by sequencing. The constructed adenovirus was generated according to the manufacturer's instructions, and virus titer was measured using Adeno-X Rapid Titer kits (BD Bioscience). And to combine the construction of shRNA-SIRP*α* preexperiment, we analyzed the RNAi sequences of SIRP*α* with software (Oligoengine, USA) and got the best RNAi sequences above mentioned for SIRP*α* silencing.

### 2.3. Infection of BM-Derived DCs

Mouse bone marrow- (BM-) derived DCs were prepared using the following procedure [25]. In brief, mouse BM was flushed from the hind limbs and passed through a nylon mesh. Using ammonium chloride, the red cells were depleted. The cells were then washed and cultured with RPMI-1640 supplemented with 10% FBS, rmGM-CSF (20 ng/mL), and rmIL-4 (20 ng/mL). On the 2nd and 4th day, the supernatant was replaced with fresh medium containing rmGM-CSF and rmIL-4. All cultures were incubated at 37°C in 5% humidified CO_2_. After 7 days of culture, flow cytometric analysis demonstrated that >80% of the cells expressed DC-specific markers. The DCs were exposed to the recombinant replication-deficient adenovirus Ad-shSIRP*α* or Ad-shControl at 10.0 multiplicity of infection (MOI). After 8 h transduction, the cells were washed with PBS and further incubated in fresh tissue culture medium. In order to develop mature DCs, the immature DCs were pulsed with HPV16mE7 protein (prepared as we previously reported in Ref. [26]) at 2.5 *μ*g/mL for 6 h, followed by stimulation with LPS (1.0 ng/mL) for 24 h. The phenotypes of the harvested DCs were tested by flow cytometry analysis.

### 2.4. SIRP*α* Silencing Analysis by Western Blot and Flow Cytometry

The transduced immature DCs were lysed with cell lysis buffer (0.3% NP40, 1 mM EDTA, 50 mM Tris-Cl, 2 mM EGTA, 1% Triton X-100, 150 mM NaCl, 25 mM NaF, 1 mM Na_3_VO_3_, and 10 *μ*g/mL phenylmethylsulfonyl fluoride (PMSF)) for 30 mins on ice and then centrifuged at 12,000 rpm for 15 mins at 4°C. The protein samples were separated via SDS-PAGE and transferred onto Immobilon membranes (Millipore, MA, USA). SIRP*α* and *β*-actin proteins were identified using rabbit anti-SIRP*α* and anti-*β*-actin monoclonal antibodies (Santa Cruz Biotechnology), respectively.

### 2.5. Cytokine Release Assay

The secretion levels of IL-12p70/IL-6/TNF-*α* produced by the transduced mature DCs were quantitated by ELISA analysis (R&D Systems Inc., Minneapolis, MN) according to the manufacturer's instructions.

### 2.6. Cytotoxicity Assay

The transduced DCs (2 × 10^5^/mouse) were subcutaneously injected into the hind foot's pads of C57BL/6 mice once a week for three weeks. Four weeks after DC inoculation, the mice were euthanized via cervical dislocation. The spleens of each group were sterilely collected. Single-cell suspensions of the pooled spleens were prepared and cultivated in CTL medium composed of RPMI-1640 which was supplemented with 10% FCS, 2 mM L-glutamine, 1 mM sodium pyruvate, 50 *μ*M 2-mercaptoethanol, and 50 *μ*g/mL gentamicin sulfate. The splenocytes as effectors were harvested after 3-day incubation and then analyzed for cytolytic activity and IFN-*γ* production. Using a CCK8 kit (Dojindo Molecular Technologies, Inc., Kumamoto, Japan), the *in vitro* cytotoxicity of splenocytes was assayed by culturing the effectors with target TC-1 cells expressing HPV16E6E7 proteins for 24 h at effector : target (*E* : *T*) ratios of 90 : 1, 30 : 1, and 10 : 1. The groups comprising a mixture of cell types were the experimental groups, whereas the control groups contained only one cell type of the TC-1 cells, splenocytes, or 1640 RPMI culture medium. The CCK8 assay was performed in 96-well plates in triplicate, and optical density (OD) was read at 570 nm. At the same time, the cocultured supernatants were collected for IFN-*γ* analysis using an IFN-*γ* ELISA kit (eBioscience Co.). In addition, 5 C57BL/6 mice were injected with sufficient anti-CD8 monoclonal antibodies (0.125 mg each) through the tail vein 1 day before DC inoculation to prove the antitumor effect of Ad-shSIRP*α*-silenced DCs. The *in vitro* cytolytic activity of splenocytes was also analyzed.

### 2.7. IFN-*γ* ELISPOT Assay

The 96-well nitrocellulose-base plates were coated with anti-mouse IFN-*γ* antibody overnight at 4°C and then blocked with complete media. The prepared splenocytes were seeded in the wells (5 × 10^5^ cells/well). PMA (5 ng/mL, Sigma) served as a positive control, and the culture media served as a negative control. Cells in a dilution series were either unstimulated or stimulated with E7.49-57 or R187 peptide (Shanghai Shenggong Biotech.) (1 *μ*g/mL). The plate was incubated overnight at 37°C/5% CO_2_ and then detected by biotinylated anti-mouse IFN-*γ* antibody for 2 h at room temperature. After removing the unbound detection antibody, streptavidin-HRP was added. The unbound streptavidin-HRP was washed off after 1 h incubation, and the plate was stained with an AEC substrate solution for 20 mins. The plate was washed and air-dried overnight. Foci of staining were counted.

### 2.8. *In Vivo* Antitumor Analysis

Female C57BL/6 (H-2^b^) mice (10 per group) received a subcutaneous (s.c.) tumor injection of 1 × 10^6^ TC-1 cells constitutively expressing wild-type HPV16E6E7. On day 9, when all mice had palpable tumors, 1 × 10^6^ prepared DCs were injected into the hind footpads of the tumor-bearing C57BL/6 mice once a week for 2 weeks. The immunized mice were intraperitoneally treated with LPS three times on days 1, 3, and 5 after DC injection. The tumor volume was calculated using the following formula (major axis × minor axis^2^) × 0.5 and recorded every three days. The tumor-bearing mice were euthanized when the tumor volume reached ~1,500 mm^3^.

### 2.9. Statistical Analysis

All data were expressed as the mean ± standard deviation (SD) and were representative of at least two different repeats. The statistical significance of group differences was measured by Student's *t*-test. *p* value < 0.05 was considered to be significant (StatXact4 software, Cytel Corporation, Cambridge, MA).

## 3. Results

### 3.1. Inhibition of SIRP*α* Expression

**Figure 1:**
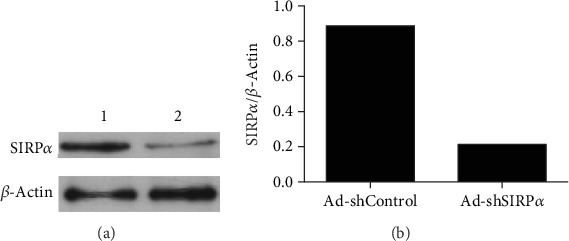
The analysis of SIRP*α* inhibition. The SIRP*α* inhibition efficacy mediated by recombinant adenovirus was analyzed by Western blot, and the results were quantified by ImageJ. Lane 1: Ad-shControl (MOI 10.0); lane 2: Ad-shSIRP*α* (MOI 10.0).

The interference efficacy of Ad-shSIRP*α* was verified by Western blot analysis, and the results were quantified by ImageJ. The Ad-shSIRP*α* infection at MOI 10.0 greatly reduced SIRP*α* expression relative to Ad-shControl (Figures 1(a) and 1(b)).

### 3.2. Phenotype Analysis of DC Maturation

The immature DCs generated from mouse bone marrow were infected with Ad-shSIRP*α* or Ad-shControl. The DC surface markers of CD80, CD83, CD86, CD1a, and HLA-DR expression levels were analyzed using flow cytometry before and after 24 h LPS stimulation. Results in Table 1 presented that the expression levels of all the antigens significantly increased by 2-4-folds in experimental groups during maturation with respect to the control. The expression levels of CD83 and CD86 increased about 10% in the Ad-shSIRP*α* group compared with the LPS group or Ad-shControl group.

### 3.3. Cytokine Release Analysis

**Figure 2:**
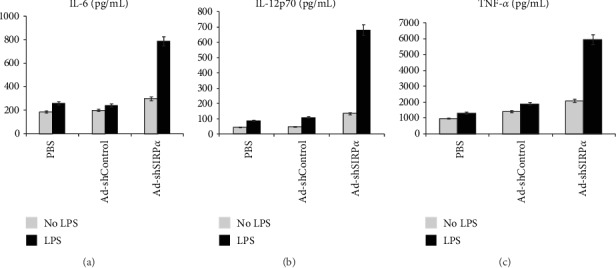
Cytokine secretion analysis. Cytokines of TNF-*α*, IL-12, and IL-6 produced by SIRP*α*-silenced DCs with or without LPS stimulation were quantitated by ELISA. Silencing SIRP*α* in DCs drastically enhanced such cytokine production in the presence or absence of LPS stimulation. Data are representative of three independent assays. *p* < 0.01 versus Ad-shControl transduced DCs.

Cytokines of TNF-*α*, IL-12, and IL-6 produced by HPV16mE7-pulsed DCs with or without LPS stimulation were quantitated by ELISA (Figures 2(a)–2(c)). Silencing SIRP*α* in DCs drastically enhanced such cytokine production in the presence or absence of LPS stimulation. By contrast, the PBS control group and the Ad-shControl group expressed far less cytokines with or without LPS stimulation.

### 3.4. Cytotoxicity Assay

**Figure 3:**
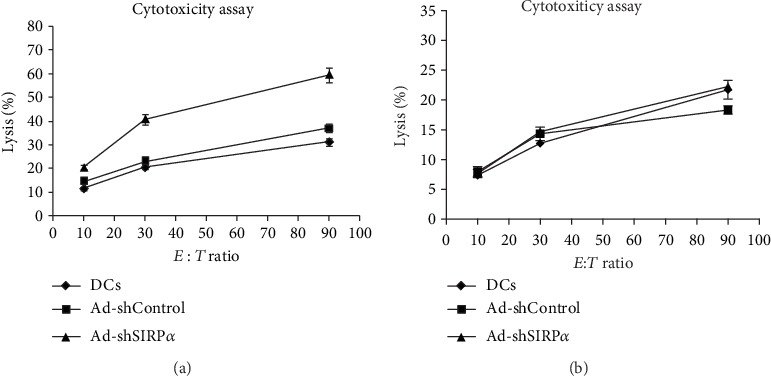
Cytotoxicity assay. The DC-induced CTL response against the HPV16E7-expressing tumor was determined using a CCK8 kit. After 2 weeks of immunization, the pooled splenocytes from each group were used as effector cells. Their specific lytic activities against TC-1 cells at 90 : 1, 30 : 1, and 10 : 1 *E* : *T* ratios were assayed. Ad-shSIRP*α*-transduced DCs induced the strongest specific CTL responses against TC-1 cells compared with the Ad-shControl group. The lytic activities against TC-1 of Ad-shSIRP*α*-transduced DCs were similar to those of the Ad-shControl group after CD8^+^T cell elimination with anti-CD8 monoclonal antibody.

2 × 10^5^ HPV16mE7-pulsed and SIRP*α*-silenced DCs were injected into the footpads of C57BL/6 mice once a week for two weeks. The DC-induced CTL response against the HPV16E7-expressing tumor was determined using a CCK8 kit. After 2 weeks of immunization, the pooled splenocytes from each group were used as effectors. Their specific lytic activities against TC-1 cells at 90 : 1, 30 : 1, and 10 : 1 *E* : *T* ratios were assayed. Ad-shSIRP*α*-transduced DCs induced the strongest specific CTL responses against TC-1 cells compared with the Ad-shControl group (Figure 3(a)). In addition, the lytic activities against TC-1 of Ad-shSIRP*α*-transduced DCs were weakened after CD8^+^T cell elimination with anti-CD8 monoclonal antibody in mice. These also indicated that Ad-shSIRP*α* knockdown DC-induced antigen processing was to activate specific CD8^+^T cells against tumor activity (Figure 3(b)). However, the effects of other immune cells may be also included.

### 3.5. ELISA and ELISPOT Analysis of IFN-*γ*

**Figure 4:**
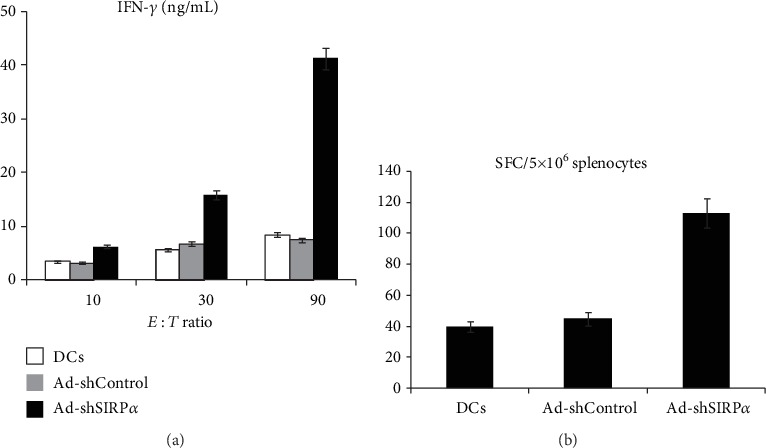
ELISA and ELISPOT analysis of IFN-*γ*. The IFN-*γ* secretion levels of the DCs induced and TC-1 cell-stimulated splenocytes at 90 : 1, 30 : 1, and 10 : 1 *E* : *T* ratios were determined by ELISA. The plate was processed according to the manufacturer's protocol, and the wells were photographed using a micropublisher camera on a stereo microscope. The dots were counted manually using an ImageJ cell counter. The frequencies of the activated antigen-specific cytotoxic T cells in splenocytes harvested from immunized C57BL/6 mice (*n* = 3) were evaluated via ELISPOT. *p* < 0.01 (Ad-shSIRP*α* vs. Ad-shControl).

The IFN-*γ* secretion levels of the DCs induced and TC-1 cell-stimulated splenocytes at 90 : 1, 30 : 1, and 10 : 1 *E* : *T* ratios were determined by ELISA. Results showed that the expression level of IFN-*γ* was greatly enhanced by silencing SIRP*α*, whereas similar effect was not observed in Ad-shControl and nontreated DCs (Figure 4(a)). The frequencies of the activated antigen-specific cytotoxic T cells in splenocytes harvested from immunized C57BL/6 mice were evaluated via ELISPOT (Figure 4(b)).

### 3.6. *In Vivo* Antitumor Activity Analysis

**Figure 5:**
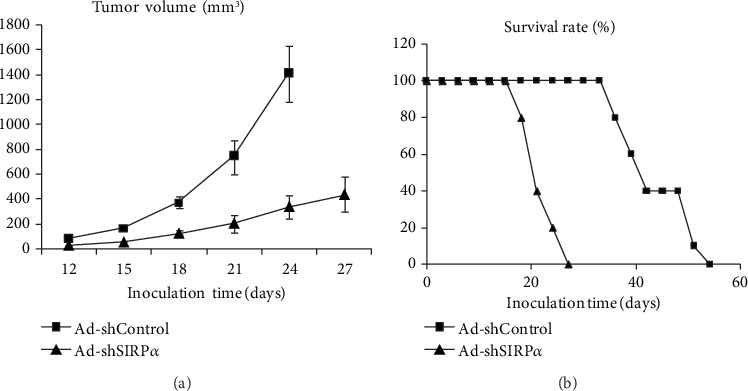
*In vivo* antitumor activity analysis. The therapeutic effect of SIRP*α*-silenced DCs was evaluated in cervical tumor xenografted C57BL/6 mice. Data are representative of three independent assays. The tumor volume after inoculation of TC-1 cells was analyzed using Student's *t*-test. *p* < 0.01 (Ad-shSIRP*α* vs. Ad-shControl). Survival rates were analyzed using the Kaplan-Meier method (log-rank test). *p* < 0.01 (Ad-shSIRP*α* vs. Ad-shControl).

1 × 10^6^ TC-1 cells constitutively expressing wild-type HPV16E6E7 were subcutaneously injected into the female C57BL/6 mice to establish homograft cervical tumor models. When all mice had palpable tumors on day 9, they were assigned to 2 groups of Ad-shSIRP*α* and Ad-shControl (10 in each group). Therapeutic treatments were initiated with a total of 2.0 × 10^6^ DCs administered intravenously into the tail of the mice for a total of 2 injections. As shown in Figure 5(a), the mice treated with Ad-shSIRP*α*-infected DCs exhibited prolonged survival time more than 40 days (40%), compared with the mice treated with Ad-shControl (0%). And the Ad-shSIRP*α*-induced DCs significantly inhibited tumor growth compared with the Ad-shControl group by which progressive tumor growth was observed (Figure 5(a)). Although therapeutic Ad-shSIRP*α* DCs could not always eradicate the tumor completely, the survival time was significantly prolonged more than 40 days in 40% of the treated mice compared with the mice in the PBS or Ad-shControl group which were all dead in 30 days (Figure 5(b)). These results indicated a therapeutic potential of Ad-shSIRP*α* DCs against HPV16 infection-associated tumors *in vivo*.

## 4. Discussion

As the reports that the SIRP*α* antibody markedly enhanced the inhibitory effect of rituximab on the growth of tumors formed by Raji cells [27], KWAR23 as one anti-SIRP*α* antibody was also a promising candidate for combination therapies to facilitate rapid and complete elimination of tumors [28]. These studies suggested that inhibiting SIRP*α* may also be more effective in treating cancers. But the immune mechanisms and signaling pathways were unclear. Immunity results from a complex interplay between the innate and the adaptive immune systems. As central antigen-presenting cells, DCs provide an essential link between innate and adaptive immune responses. The protective antitumor immunity generated by DCs depends on their efficacy of presenting tumor antigens to induce tumor-specific effector T cells that can specifically reduce the tumor size [25]. The capability of DCs to capture, process, and present antigens depends on their differentiation/maturation stage and origin. Activated mature DCs can induce protective antitumor immunity in contrast to immature DCs that present self-antigens to T cells resulting in immune tolerance by generating suppressor T cells or T cell deletion [29–32]. T cell-mediated immunity and pathology will also bring into many spotlight potential targets for novel cancer therapies [33].

In order to activate the full immunostimulatory talent of DCs, SIRP*α* which initiated a negative signal pathway to regulate the maturation of DCs was knocked down in this research. After further pulsing with a HPV16E7 (HPV16mE7) protein with enhanced antigenicity and reduced transformation activity, TNF-*α*/IL-12/IL-6 secretion levels as well as the CTL inducing efficacy of the *ex vivo* cultured DCs were evaluated. The results demonstrated not only an improved cytokine production but also an enhanced LPS-stimulated cytokine secretion. The IFN-*γ* ELISA and ELISPOT analysis also showed an increased IFN-*γ* production. To further investigate the antineoplastic activity mediated by SIRP*α* knockdown, the HPV16mE7 loaded and *ex vivo* cultured DCs were injected into the tumor-bearing C57BL/6 mice. The T lymphocytes activated by SIRP*α*-silenced DCs significantly decreased the tumor mass compared with controls in which progressive tumor growth was observed. And CD8^+^T lymphocytes were specific and necessary in the antigen presentation processes of Ad-shSIRP*α* knockdown in DCs against tumor activity. Moreover, the survival time was significantly prolonged (60%) at day of 60 in comparison with the mice administered with Ad-shControl-treated DCs.

Taken together, these results suggest that SIRP*α*-silenced DC vaccination presented potential therapeutic implications against cervical cancer. This could serve as the basis to molecular immunotherapy that could be tailored for individual patients or different cancer types.

## 5. Conclusions

The SIRP*α* knockdown in DCs results in increased cytokine (TNF-*α*/IL-12/IL-6) secretion, IFN-*γ* secretion by T lymphocytes, and *in vitro*/*in vivo* tumoricidal activity against cervical cancer. These results suggest that SIRP*α*-silenced DC vaccination presented potential therapeutic implications against cervical cancer.

## Figures and Tables

**Table 1:** Percentage of DCs expressing CD1a, CD80, CD83, CD86, and HLA-DR.

	CD1a	CD80	CD83	CD86	HLA-DR
Control	27.1 ± 5.6	31.6 ± 3.4	33.2 ± 5.3	47.5 ± 2.9	54.6 ± 4.3
LPS	91.5 ± 3.2^a^	89.4 ± 6.4^a^	72.8 ± 8.3^a^	81.3 ± 5.4^a^	96.2 ± 2.2^a^
Ad-shControl	92.1 ± 2.9^a^	87.4 ± 7.3^a^	74.6 ± 7.6^a^	80.7 ± 6.2^a^	97.3 ± 1.1^a^
Ad-shSIRP*α*	94.5 ± 2.1^a,b^	90.4 ± 3.1^a,b^	92.3 ± 4.8^a,c^	95.2 ± 1.9^a,c^	96.3 ± 2.1^a,b^

Immature DCs were stimulated by Ad-shSIRP*α*. Percent of DCs expressing CD1a, CD80, CD83, CD86, and HLA-DR was detected using flow cytometry before and after 24 h TNF-*α* stimulation and analyzed by one-way ANOVA. ^a^Significant differences compared with that of the DC control, *p* < 0.001; ^b^no significant differences compared with that of the LPS control, *p* > 0.05; ^c^significant differences compared with that of the LPS control or Ad-shControl, *p* < 0.05.

## Data Availability

The data used to support the findings of this study are available from the corresponding authors upon request.

## Abbreviations

SIRP*α*: Signal regulatory protein *α*
DCs: Dendritic cells
APC: Antigen-presenting cells
STAT3: Signal transducer and activators of transcription-3
ATCC: American Type Culture Collection
BM: Bone marrow
PMA: Phorbol 12-myristate 13-acetate
AEC: 3-Amino-9-ethylcarbazole.
